# Opposite Expression of SPARC between the Liver and Pancreas in Streptozotocin-Induced Diabetic Rats

**DOI:** 10.1371/journal.pone.0131189

**Published:** 2015-06-25

**Authors:** Kanikkai Raja Aseer, Sang Woo Kim, Myung-Sook Choi, Jong Won Yun

**Affiliations:** 1 Department of Biotechnology, Daegu University, Kyungsan, Kyungbuk, 712–714, Republic of Korea; 2 Center for Food and Nutritional Genomics Research & Department of Food Science and Nutrition, Kyungpook National University, Daegu, 702–701, Republic of Korea; Chi-Mei Medical Center, TAIWAN

## Abstract

Secreted protein acidic and rich in cysteine (SPARC) is a matricellular protein that regulates several cellular events, including inflammation and tissue remodelling. In this study, we investigated the tissue-specific expression of SPARC in streptozotocin (STZ)-induced diabetes, and found that SPARC was significantly up-regulated in the liver while down-regulated in the pancreas of STZ-induced diabetic rats. Chronic inflammation occurred in the diabetic pancreas accompanied by up-regulation of CCAAT/enhancer-binding protein beta (C/EBPβ) and its targets (TNFα, *Il6*, CRP, and *Fn1*) as well as myeloperoxidase (*Mpo*) and C-X-C chemokine receptor type 2 (*Cxcr2*). Diabetic liver showed significant up-regulation of *Tgfb1* as well as moderately less up-regulated TNFα and reduced *Fn1*, resulting in elevated fibrogenesis. PARP-1 was not up-regulated during CD95-mediated apoptosis, resulting in restoration of high ATP levels in the diabetic liver. On the contrary, CD95-dependent apoptosis was not observed in the diabetic pancreas due to up-regulation of PARP-1 and ATP depletion, resulting in necrosis. The cytoprotective machinery was damaged by pancreatic inflammation, whereas adequate antioxidant capacity indicates low oxidative stress in the diabetic liver. High and low cellular insulin content was found in the diabetic liver and pancreas, respectively. Furthermore, we identified six novel interacting partner proteins of SPARC by co-immunoprecipitation in the diabetic liver and pancreas, and their interactions with SPARC were predicted by bioinformatics tools. Taken together, opposite expression of SPARC in the diabetic liver and pancreas may be related to inflammation and immune cell infiltration, degrees of apoptosis and fibrosis, cytoprotective machinery, and cellular insulin levels.

## Introduction

Type 1 diabetes mellitus (T1DM) results from the autoimmune destruction of insulin-producing β-cells in the pancreas, leading to dysregulation of blood glucose levels [1]. Streptozotocin (STZ)-induced animal models have been widely used in medical research to understand the pathophysiology of T1DM based on the ability of STZ to disrupt pancreatic β-cells [2]. In rat models of T1DM, pancreatic tissue shows gender-based distinctions in islet size and number as well as the area occupied by β-cells, which are larger in females as compared to males [3]. Nevertheless, female rats are less sensitive to insulin than male rats and more susceptible to the severe form of diabetes [3]. On the other hand, hepatic tissue in female rats is known to display elevated protein content, phosphorylation, and oxidative capacity compared to male counterparts [4].

In our previous studies, we showed that several regulatory proteins in STZ-induced diabetic rats play key regulatory roles in a sex- and tissue-specific manner [5–7]. Among these proteins, secreted protein acidic and rich in cysteine (SPARC) attracted our attention in the context of T1DM because we previously reported that SPARC might play a vital role in resistance to obesity development [8]. SPARC, also known as osteonectin and BM40, is a secreted protein found in the extracellular matrix (ECM) [9]. SPARC is expressed during tissue remodelling and repair [9] and is involved in the development of several cancers [10]. Recently, SPARC has been suggested as a key player in pathologies associated with diabetic complications such as retinopathy and nephropathy [11, 12]. Further, SPARC-knockout mice are protected from type 2 diabetes mellitus (T2DM) and its complications, supporting a role for SPARC in causing T2DM [13]. In particular, elevated SPARC levels have been proposed to contribute to the pathogenesis of obesity and T2DM by promoting insulin resistance [14, 15]. Nonetheless, it remains to be determined whether SPARC plays a role in T1DM. The STZ-induced onset of diabetes-related kidney growth is associated with reduction of SPARC protein expression [16]. In contrast, increased expression of SPARC has been observed in mesenteric vessels of STZ-induced diabetic rats, suggesting pathological significance in the development of vascular remodelling in diabetes [17]. These findings strongly suggest that SPARC may play organ-specific roles in diabetes. SPARC is also constitutively expressed in the liver and shows elevated expression during fibrosis [18]. In addition, STZ treatment is involved in accelerated liver fibrosis [19]. An earlier report has shown that knockdown of hepatic SPARC expression improves thioacetamide-induced liver fibrosis in rats with chronic liver injury [18].

Although SPARC is predominantly expressed in adipose tissue, it has also been detected in the pancreas [20]. Recent studies have demonstrated that SPARC regulation is involved in maintenance of β-cell function [21] and islet cell survival [22]. Expression of SPARC is increased in pancreatitis and decreased upon destruction of pancreatic acinar cells [20]. However, no studies have examined whether SPARC expression is associated with impaired insulin secretion. Moreover, pathophysiological roles of SPARC in the diabetic liver have not been demonstrated.

In this study, we found that SPARC was regulated in a tissue-specific manner in STZ-induced diabetic rats, and it showed opposite expression patterns in the liver and pancreas of diabetic rats. Therefore, we tried to elucidate disparity in the expression of SPARC along with its interacting protein partners in the diabetic liver and pancreas of both genders. To the best of our knowledge, the present study is the first to demonstrate the tissue-specific regulation of SPARC along with its novel interactive partner proteins in the liver and pancreas of STZ-induced diabetic rats.

## Materials and Methods

### Ethics statement

The Daegu University Animal Care and Use Committee approved this study according to Guide for the Care and Use of Laboratory Animals published by the National Institutes of Health.

### Animal breeding and diabetes induction

Male and female Sprague-Dawley (SD) rats (11 weeks of age) were purchased from Daehan Experiment Animals (Hanam, Korea), and all rats were housed under standard conditions (temperature 22°C, 12 h light/dark cycle, relative humidity 55%) with free access to water and food pellets (Feed Korea Lab, Hanam, Korea). After 1 week of acclimatization, 48 animals were subdivided into four groups: male control (*n* = 8), male STZ (*n* = 16), female control (*n* = 8), and female STZ (*n* = 16). For induction of experimental diabetes, food was removed overnight, after which male and female rats received a single intravenous injection of STZ (50 mg/kg of STZ in 0.01 M sodium citrate buffer, pH 4.5) and food was returned 4 h later. Rats in the control groups were injected in parallel with citrate buffer alone. Body weight as well as blood glucose and insulin levels were determined for the 2-week period at the same time point. Blood glucose was measured by using glucose oxidase reagent strips and a compact glucometer (LifeScan, Inc., Milpitas, CA, USA), and a blood glucose level of ≥300 mg/dL indicated successful induction of diabetes. Based on the measured glucose levels, six rats from each group were selected. Body weights as well as blood glucose and insulin levels were checked periodically on days 0, 7, 10, and 14. Animals were sacrificed on day 14, after which the liver and pancreas were dissected, snap-frozen in liquid nitrogen, and stored at -80°C until subjected to RNA and protein isolation. Plasma insulin levels were measured using a sandwich enzyme-linked immunosorbent assay (ELISA) system, including Rat Insulin kit (ALPCO, Salem, NH, USA). Each assay was performed in triplicate using individual plasma samples from six selected rats per group according to the manufacturer’s protocol.

### Total RNA preparation and quantitative real-time RT-PCR

Total RNA was isolated using a total RNA isolation kit (RNA-spin, iNtRON Biotechnology, Seongnam, Korea) from each tissue sample group. RNA (1 μg) was transcribed to cDNA by using Maxime RT premix (iNtRON Biotechnology), and the resulting cDNA was quantitatively measured by employing Fast start universal SYBR Green mastermix (Roche, Basel, Switzerland) using real-time RT-PCR (Stratagene 246 mx 3000p QPCR System, Agilent Technologies, Santa Clara, CA, USA). Each run was evaluated in triplicate, and genes of interest were normalized to levels of *B2m* (β-2 microglobulin-encoding gene) and *Ppib* (peptidylprolyl isomerase B-encoding gene). Sequences of primer sets used in this study are listed in Table 1.

**Table 1:** Sequences of primers used for quantitative real-time PCR in this study.

Gene	Forward primer	Reverse primer
*Sparc*	CCACTCGCTTCTTTGAGACC	TAGTGGAAGTGGGTGGGGAC
*Mpo*	ACCTACCCCAGTACCGATCC	AACTCTCCAGCTGGCAAAAA
*Il1b*	CCCTGCAGCTGGAGAGTGTGG	TGTGCTCTGCTTGAGAGGTGCT
*Il6*	CGAGCCCACCAGGAACGAAAGTC	CTGGCTGGAAGTCTCTTGCGGAG
*Cxcr2*	CCATCTTCATTCTTCGGACT	AACAGGACAATGTTGTAGGGA
*Tgfb1*	GAGGTGACCTGGGCACCATCCATGAC	CTGCTCCACCTTGGGCTTGCGACCCC
*Fn1*	AGCCCGGATGTCAGAAGCTATAC	AGCGTGTACAGGTGGATCTTG
*B2m*	GTGTCTCAGTTCCACCCACC	GGTGTGAATTCAGTGTGAGCC
*Ppib*	GCCTCTCGGAGCGCAATATG	CTTATCGTTGGCCACGGAGG

### Immunoblot analysis

Hepatic and pancreatic tissue lysates from control and STZ-induced male and female rats containing equal amounts of protein were separated by SDS-PAGE. After electrophoresis, proteins were transferred to polyvinylidene difluoride (PVDF) membranes, blocked for 1h with 5% skim milk at room temperature, and incubated with the indicated primary antibodies for 2 h at 1:1000 dilution (anti-β-actin, anti-SPARC, anti-C/EBPβ, anti-ATP5B, anti-CD95, anti-MCP1, anti-iNOS, anti-Cyt C, anti-SOD2, anti-INS, anti-CA3, anti-RARhoGAP, anti-CPS1, anti-BHMT, anti-PA, anti-APC2 [Santa Cruz Biotechnology, Santa Cruz, CA, USA], anti-TNFα, anti-PARP-1, anti-NFκB, anti-HSP90 [Cell Signaling Technology, Beverly, MA, USA], and anti-CRP [AbFrontier, Seoul, Korea]). After washing with Tris-buffered saline containing Tween 20, the membranes were incubated with HRP-conjugated secondary antibodies for 1 h at room temperature. Then, immune complexes were detected using the ECL method, and immunoreactive bands were quantified by densitometric analysis using ImageMaster 2D software version 4.95 (GE Healthcare, Little Chalfont, Buckinghamshire, UK). Relative intensity (%) values of proteins were normalized to β-actin levels.

### Histological and immunohistochemical analyses

For the histological study, pancreatic and hepatic tissues were fixed in 10% neutral-buffered formalin and embedded in paraffin wax. Paraffin-embedded tissue sections (4 μm each) were deparaffinized, rehydrated, and subjected to hematoxylin and eosin staining. Immunohistochemistry was performed on formalin fixed, paraffin-embedded hepatic and pancreatic tissues. The sections were then incubated with primary antibodies (anti-SPARC, anti-CA3, anti-CPS1, anti-RARhoGAP, anti-BHMT, anti-PA, anti-APC2 [dilution 1:100, Santa Cruz Biotechnology], and anti-insulin [dilution 1:400, Cell Signaling Technology]) overnight at 4°C, followed by incubation with appropriate HRP-conjugated secondary antibodies at room temperature for 2 h. The immunoreactivity was visualized with diaminobenzidine (DAB) staining (Vector Laboratories, Burlingame, CA, USA), and then counterstained with Mayer's hematoxylin (Vector Laboratories). The histopathological findings were observed using light microscopy (X400; Olympus IX51, Tokyo, Japan).

### Identification of SPARC-interacting partner proteins by Co-Immunoprecipitation (Co-IP)

Co-IP was performed in order to isolate the immune complexes associated with SPARC in the liver and pancreas. Briefly, pooled tissue lysates containing 300 mg of proteins were incubated with 2.5 μg of anti-SPARC monoclonal antibody (Santa Cruz Biotechnology) at 4°C for 16 h and then, 20 μl of A/G PLUS agarose beads (Santa Cruz Biotechnology) was added and incubated at 4°C for 12 h. The immunoprecipitates were collected, washed, and denatured with 2X Laemmli buffer and then resolved by 10% SDS-PAGE. Rabbit and mouse IgGs (Santa Cruz Biotechnology) were used to control non-specific antibody interactions. After silver staining, we detected a total of 18 and 14 protein bands from liver and pancreas, respectively. Each protein band was excised from the gel, digested with trypsin (Promega, Madison, WI), mixed with α-cyano-4-hydroxycinnamic acid in 50% acetonitrile/0.1% TFA, and subjected to MALDI-TOF analysis (Microflex LRF 20, Bruker Daltonics, Billerica, MA, USA), as previously described [23]. Spectra were collected from 300 shots per spectrum over m/z range 600–3000 and calibrated by two point internal calibration using trypsin auto-digestion peaks (m/z 842.5099, 2211.1046). Peak list was generated using Flex Analysis 3.0. Threshold used for peak-picking was as follows: 500 for minimum resolution of monoisotopic mass, 5 for S/N. The search program MASCOT, developed by the Matrix Science (http://www.matrixscience.com), was used for protein identification by peptide mass fingerprinting (PMF). We were able to identify four proteins in liver and two proteins in pancreas as SPARC-interacting partners. The following parameters were used for the database search: trypsin as the cleaving enzyme, a maximum of one missed cleavage, iodoacetamide (Cys) as a complete modification, oxidation (Met) as a partial modification, monoisotopic masses, and a mass tolerance of ± 0.1 Da. The MASCOT probability based MOWSE (molecular weight search) score was calculated for PMF. The protein score was -10*Log (*p*), where *p* was the probability that the identified match was a random event and greater than 61 was considered significant (*p*<0.05). Accession number, nominal mass, calculated pI, sequence coverage, and MASCOT score for the identified SPARC-interacting proteins are given in Table 2.

**Table 2:** Identified SPARC-interacting partner proteins in liver and pancreas.

Proteins	Acc. No. ^1)^	Nominal mass (Mr) ^2)^	Calculated pI	Protein sequence coverage (%)	MASCOT score ^3)^
**SPARC-interacting partner proteins identified in liver**					
Carbamoyl-phosphate synthase (CPS1)	gi|8393186	164476	6.33	15	111
Rho GTPase-activating protein 20 (RARhoGAP)	gi|564325101	43130	8.91	29	114
Betaine-homocysteine S-methyltransferase 1(BHMT)	gi|13540663	44948	8.02	39	157
Carbonic anhydrase 3 (CA3)	gi|31377484	29413	6.89	49	159
**SPARC-interacting partner proteins identified in pancreas**					
Pancreatic alpha-amylase (PA)	gi|564338673	57093	7.92	45	206
Adenomatous polyposis coli protein 2 (APC2)	gi|158186657	243779	8.97	4	63

^1)^ Acc. No. is a NCBInr database accession number.

^2)^ The nominal mass is the integer mass of the most abundant naturally occurring stable isotope of an element. The nominal mass of a molecule is the sum of the nominal masses of the elements in its empirical formula.

^3)^ MASCOT probability-based MOWSE (molecular weight search) score calculated for PMF. Protein score is -10*Log (*P*), where *P* is the probability that the observed match is a random event and greater than 61 are significant (*p*<0.05).

### SPARC-interacting protein networks and pathway analysis

We performed bioinformatics analysis using Search Tool for the Retrieval of Interacting Genes (STRING) version 9.1 (http://string-db.org) in order to predict physical protein-protein interaction networks [24] for the SPARC-interacting protein partners of liver (BHMT/*Bhmt*, CA3/*Car3*, CPS1/*Cps1*, and RARhoGAP/ARHGAP20/*Arhgap20*, and pancreas (PA/AMY2/*Amy2* and APC2/*Apc2*). The predicted protein-protein interactions are based on experimental and curated databases, and the protein interactions can be displayed according to their confidence, evidence, or actions. In addition, we conducted functional bioinformatics analysis using Ingenuity Pathway Analysis (IPA, QIAGEN Redwood City, www.qiagen.com/ingenuity) in order to generate tissue-specific functional networks. We subjected the molecular networks that were built by Ingenuity to functional analysis. As a result, IPA assigns bio-functional categories/cellular functions to the respective molecular networks. Based on statistical significance and biological importance, IPA enables functional analysis of a protein dataset in the context of molecular, cellular, disease/functions from curated biological interactions and functional annotations found in Ingenuity Knowledge Base and external databases. This facilitated identification of cellular functions significantly represented by SPARC and its interacting proteins in the liver and pancreas. Applying biological filters is an advantage of IPA to focus on the changes most relevant to particular tissues specific to a given species. Certainly, the options in IPA are very stringent and *p*-value is calculated with the right-tailed Fisher's Exact Test. The detailed procedure for pathway analysis using IPA was described in S1 Supporting Information.

### Statistical analysis

All data are presented as the mean ±S.D and differences between groups were determined by Student’s *t*-test or One-way Analysis of Variance (ANOVA) using the Statistical Package of Social Science (SPSS, version 17.0; SPSS Inc., Chicago, IL, USA) program, followed by Tukey’s post hoc tests. Statistical significances between gender as well as controls and diabetic rats were indicated as either *p*<0.05 or *p*<0.01.

## Results

### Diabetic phenotypes

**Fig 1:**
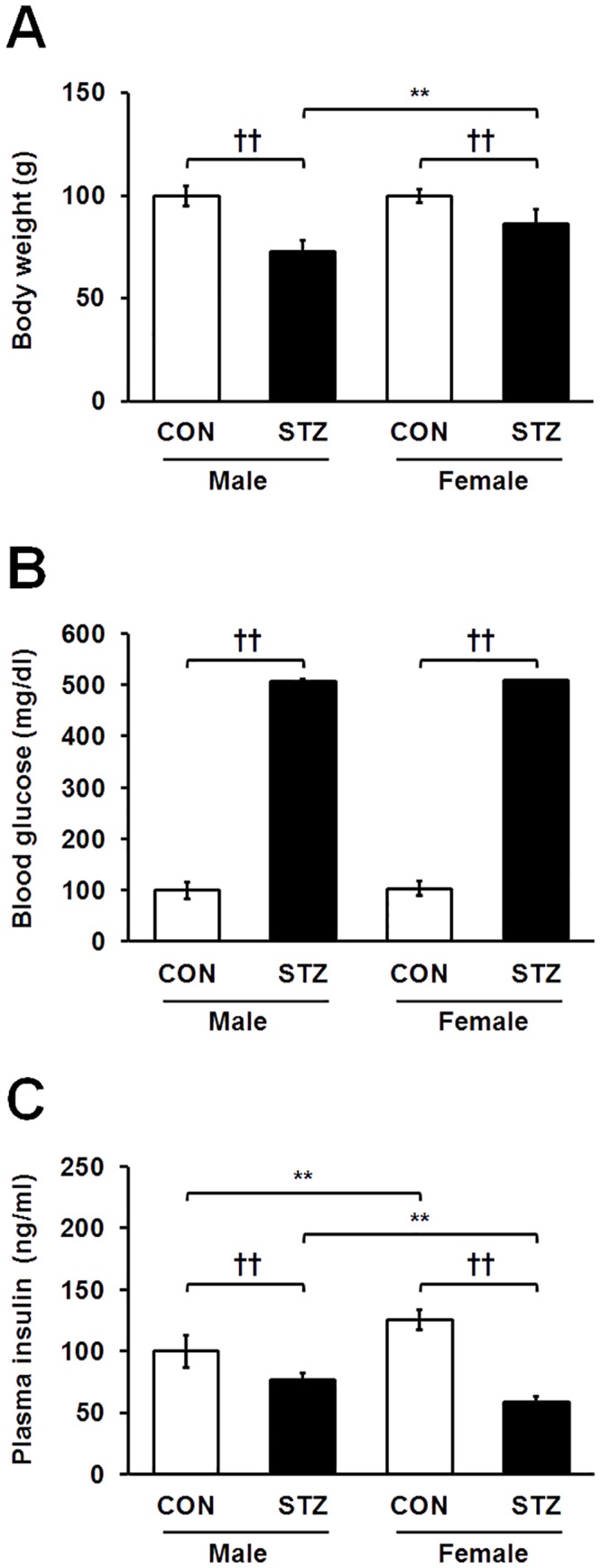
Sex-dependent changes in body weight (A), blood glucose levels (B), and plasma insulin levels (C) in healthy control and STZ-induced diabetic rats. Statistical significance between male and female rats was calculated by One-way ANOVA followed by Tukey’s post hoc tests, where *p*-value is *p<0.05 and **p<0.01, and significance between controls and diabetics is represented by ^†^ *p*<0.05 and ^††^ *p*<0.01.

To investigate the role of SPARC in T1DM, we developed an experimental diabetic rat model using STZ (50 mg/kg body weight) as outlined in our previous study [7]. We comparatively determined total body weights, blood glucose levels, and plasma insulin levels in control and STZ-induced diabetic male and female rats. As shown in Fig 1A, both diabetic rats showed reduced body weight, and the males were more significantly reduced than the females after 2 weeks of STZ treatment. Further, blood glucose levels were higher in both genders upon STZ induction, indicating insulin insufficiency (Fig 1B). Reduction of insulin levels was also observed in both genders of diabetic rats, and females showed lower insulin levels than male counterparts (Fig 1C).

### Opposite expression of SPARC in liver and pancreas of diabetic rats

**Fig 2:**
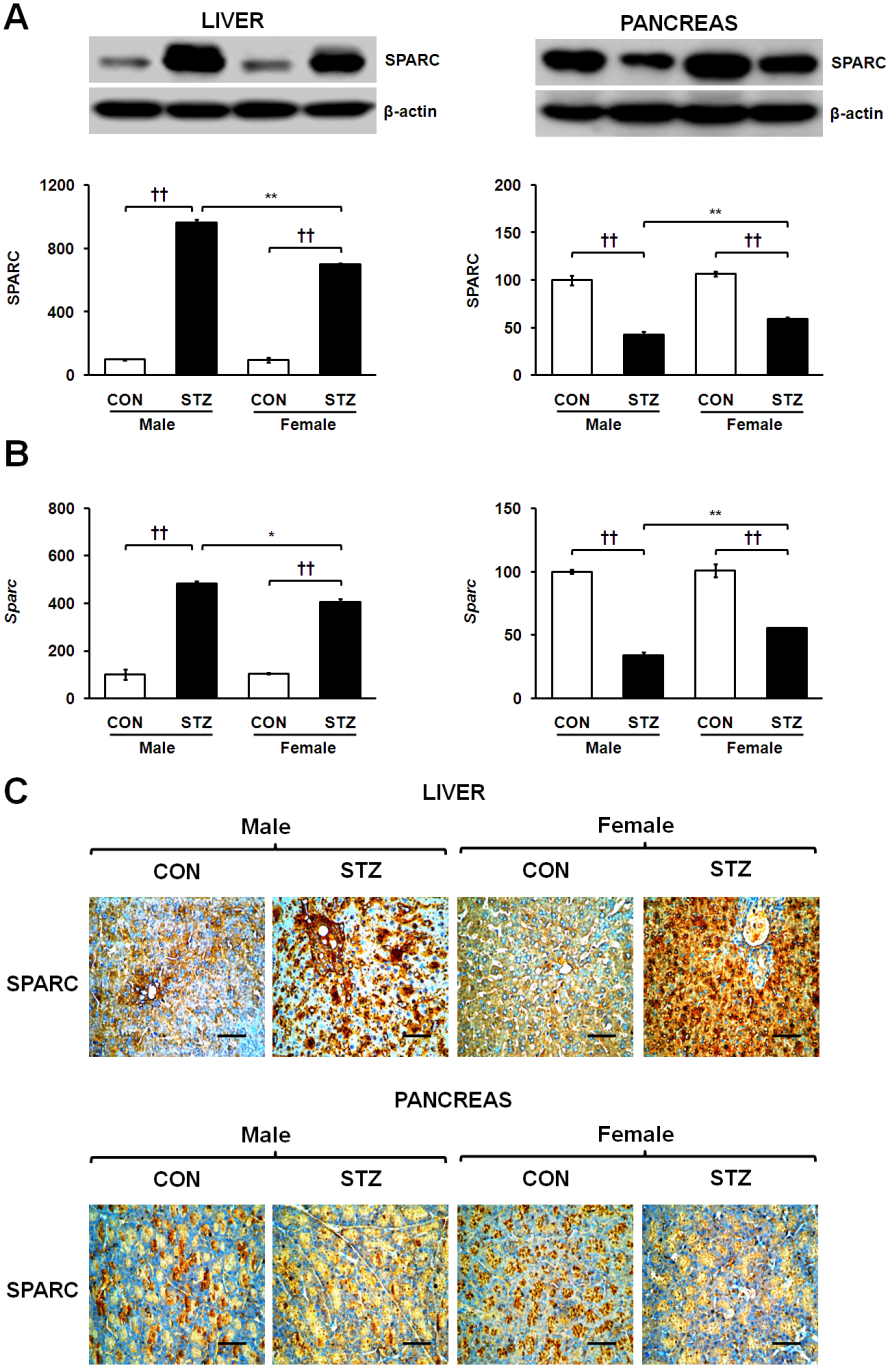
Tissue-dependent expression of SPARC in the liver and pancreas of males and females at protein (A) and mRNA levels (B). Immunohistochemistry of SPARC in hepatic and pancreatic tissues of control and STZ-induced diabetic rats (C), where representative photomicrographs are shown and sections were counter-stained with haematoxylin (magnification X400 and scale bar represents 50 μm). Data are representative of three independent experiments. Statistical significance between male and female rats was calculated by One-way ANOVA followed by Tukey’s post hoc tests, where *p*-value is *p<0.05 and **p<0.01, and significance between controls and diabetics is represented by ^†^ *p*<0.05 and ^††^ *p*<0.01.

Of primary interest, tissue-specific SPARC expression in the STZ-induced diabetic liver and pancreas was verified at the protein and mRNA levels. Interestingly, STZ induction significantly up-regulated protein and mRNA expression of SPARC in diabetic livers of both genders (Fig 2A and 2B, respectively). Of note, mRNA and protein levels of SPARC in the diabetic liver were significantly higher in male rats as compared to their female diabetic counterparts. To our surprise, the diabetic pancreas showed the opposite result, displaying markedly decreased protein and mRNA expression of SPARC in both genders, however these levels were more significantly reduced in diabetic male rats than females (Fig 2A and 2B, respectively). Consistent with our immunoblot analysis, the diabetic liver showed increased SPARC immunoreactivity, whereas diminished reactivity was observed in acinar region of the diabetic pancreas (Fig 2C). This apparent discrepancy in the regulation of SPARC expression between the diabetic liver and pancreas led us to investigate SPARC levels under STZ-induced diabetic conditions.

### Tissue-dependent regulation of CCAAT/enhancer-binding protein beta (C/EBPβ), tumor necrosis factor α (TNFα), interleukin-6 (*Il6*) and acute phase signatures in liver and pancreas of diabetic rats

**Fig 3:**
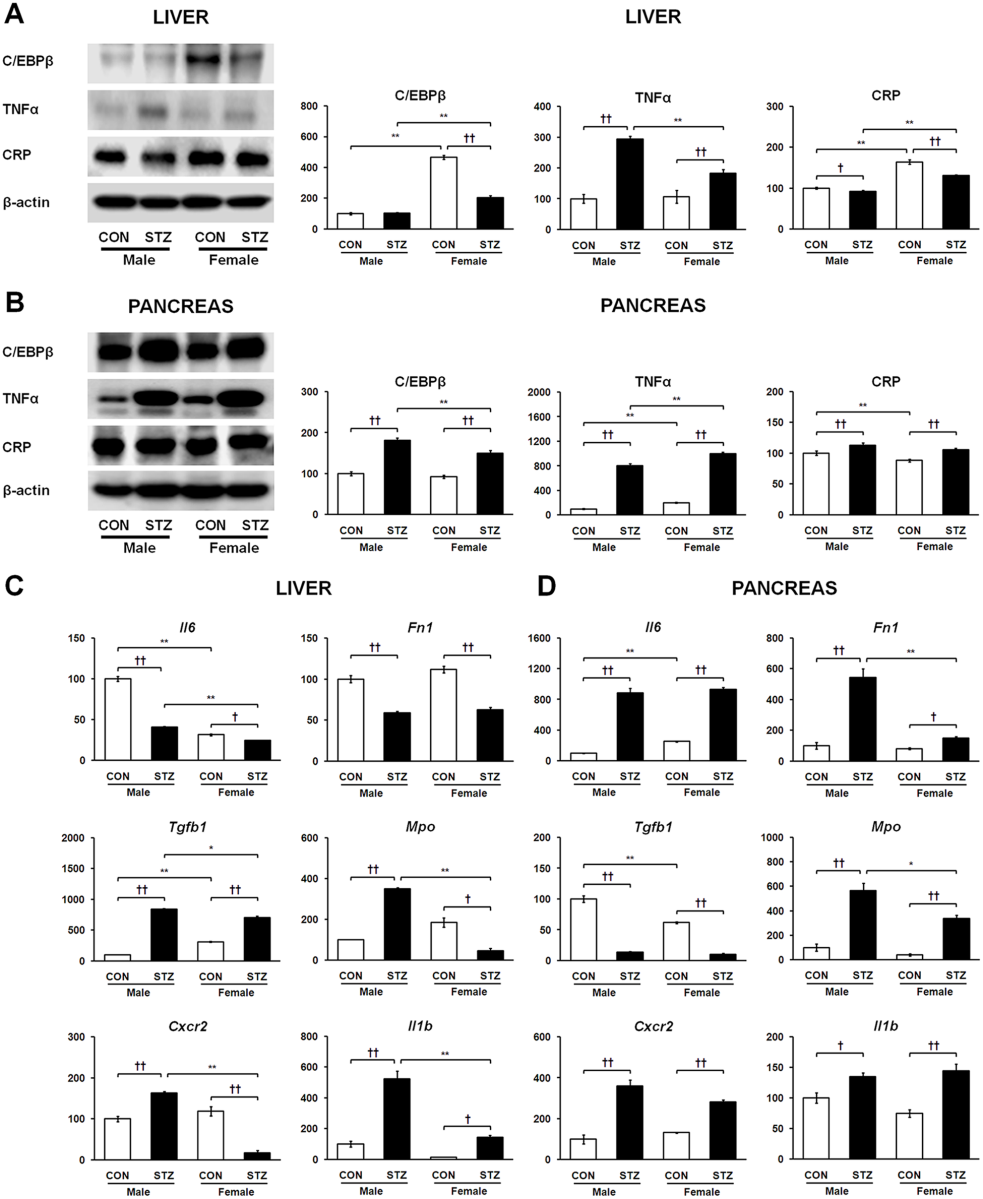
Tissue-dependent protein expression levels of C/EBPβ, TNFα, CRP, and mRNA expression levels of *Il6*, *Fn1*, *Tgfb1*, *Mpo*, *Cxcr2*, and *Il1b* in liver (A and C) and pancreas (B and D), respectively. Data are representative of three independent experiments. Statistical significance between male and female rats was calculated by One-way ANOVA followed by Tukey’s post hoc tests, where *p*-value is *p<0.05 and **p<0.01, and significance between controls and diabetics is represented by ^†^ *p*<0.05 and ^††^ *p*<0.01.

We first elucidated the correlation of immune cell infiltration and inflammatory response markers with tissue-specific expression of SPARC. To this end, we tested related major molecules on the basis of proinflammatory cytokines and acute phase molecules. We examined expression levels of C/EBPβ and its classic targets, TNFα and *Il6*, in the diabetic liver and pancreas. Specifically, as shown in Fig 3A and 3B, C/EBPβ protein was highly up-regulated in the diabetic pancreas and down-regulated in the diabetic livers of female rats with no significant changes in male rats. Interestingly, the data indicate that TNFα protein expression was moderately less up-regulated in the diabetic liver (Fig 3A) while highly up-regulated in the STZ-induced diabetic pancreas (Fig 3B). Consequently, mRNA levels of *Il6* were dramatically elevated in the diabetic pancreas as opposed to significantly reduced in the diabetic liver (Fig 3C and 3D). Regarding the direct correlation between *Il6* and its acute phase targets C-reactive protein (CRP) and fibronectin (*Fn1*), we observed their levels decreased in the diabetic liver (Fig 3A and 3C) and increased in the diabetic pancreas (Fig 3B and 3D). These results suggest that the varying expression of C/EBPβ as well as TNFα, *Il6*, CRP, and *Fn1* may show correlation with tissue-specific and opposite expression of SPARC in STZ-induced diabetic liver and pancreas.

### Tissue-specific differences in expression of transforming growth factor beta 1 (*Tgfb1*) and immune cell infiltration markers in STZ-induced diabetic liver and pancreas

**Fig 4:**
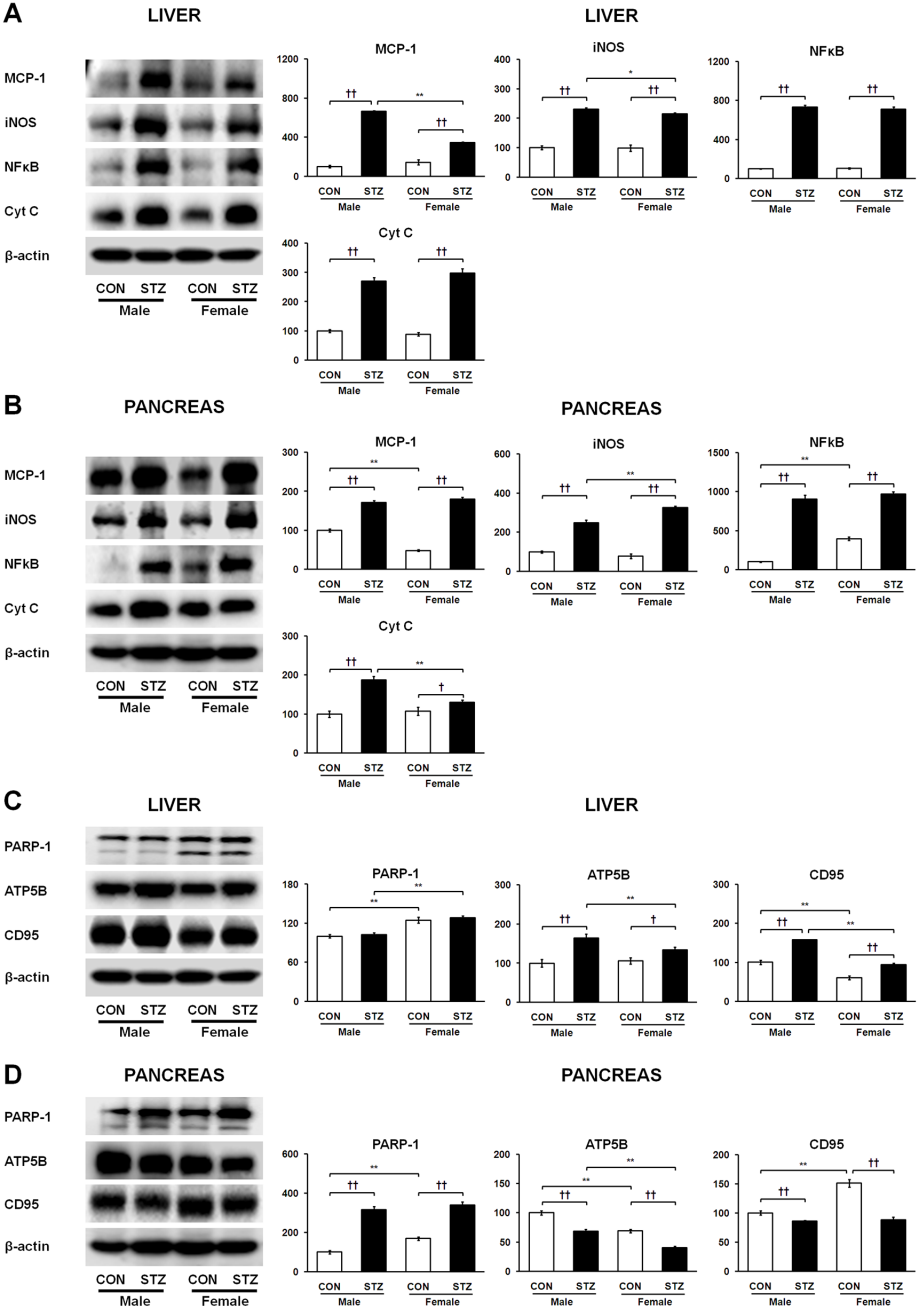
Tissue-dependent protein expression levels of MCP-1, iNOS, NFκB, Cyt C, PARP-1 ATP5B, and CD95 in liver (A and C) and pancreas (B and D), respectively. Data are representative of three independent experiments. Statistical significance between male and female rats was calculated by One-way ANOVA followed by Tukey’s post hoc tests, where *p*-value is *p<0.05 and **p<0.01, and significance between controls and diabetics is represented by ^†^ *p*<0.05 and ^††^ *p*<0.01.

We observed tissue-specific regulation of *Tgfb1* expression in the diabetic liver and pancreas. The mRNA expression of *Tgfb1* was strikingly up-regulated in the diabetic liver (Fig 3C), whereas it was significantly down-regulated in the diabetic pancreas (Fig 3D). The mRNA expression of immune cell infiltration markers myeloperoxidase (*Mpo*) and C-X-C chemokine receptor type 2 (*Cxcr2*) markedly increased in the diabetic livers of male rats. On the contrary, these levels were significantly attenuated in the livers of STZ-induced female rats and showed clear gender dimorphism (Fig 3C). The mRNA expression of *Mpo* and *Cxcr2* was highly up-regulated in the diabetic pancreas of both genders (Fig 3D). Thus, these findings may be relevant in the context of *Tgfb1* activity in infiltrating immune cells, which may be associated with opposite regulation of SPARC in the diabetic liver and pancreas. Regardless of the tissue-specific expression patterns of other inflammatory mediators, interleukin-1 beta (*Il1b*) gene, monocyte chemoattractant protein 1 (MCP-1), nitric oxide synthase (iNOS), nuclear factor kappa B (NFκB), and cytochrome C oxidase (Cyt C) were up-regulated in the diabetic liver and pancreas (Fig 3C and 3D and Fig 4A and 4B) of both genders. Remarkably, all of these proteins/genes showed relatively less up-regulation only in the diabetic liver of females as compared to diabetic male rats.

### Differences in apoptosis and necrosis between STZ-induced diabetic liver and pancreas

**Fig 5:**
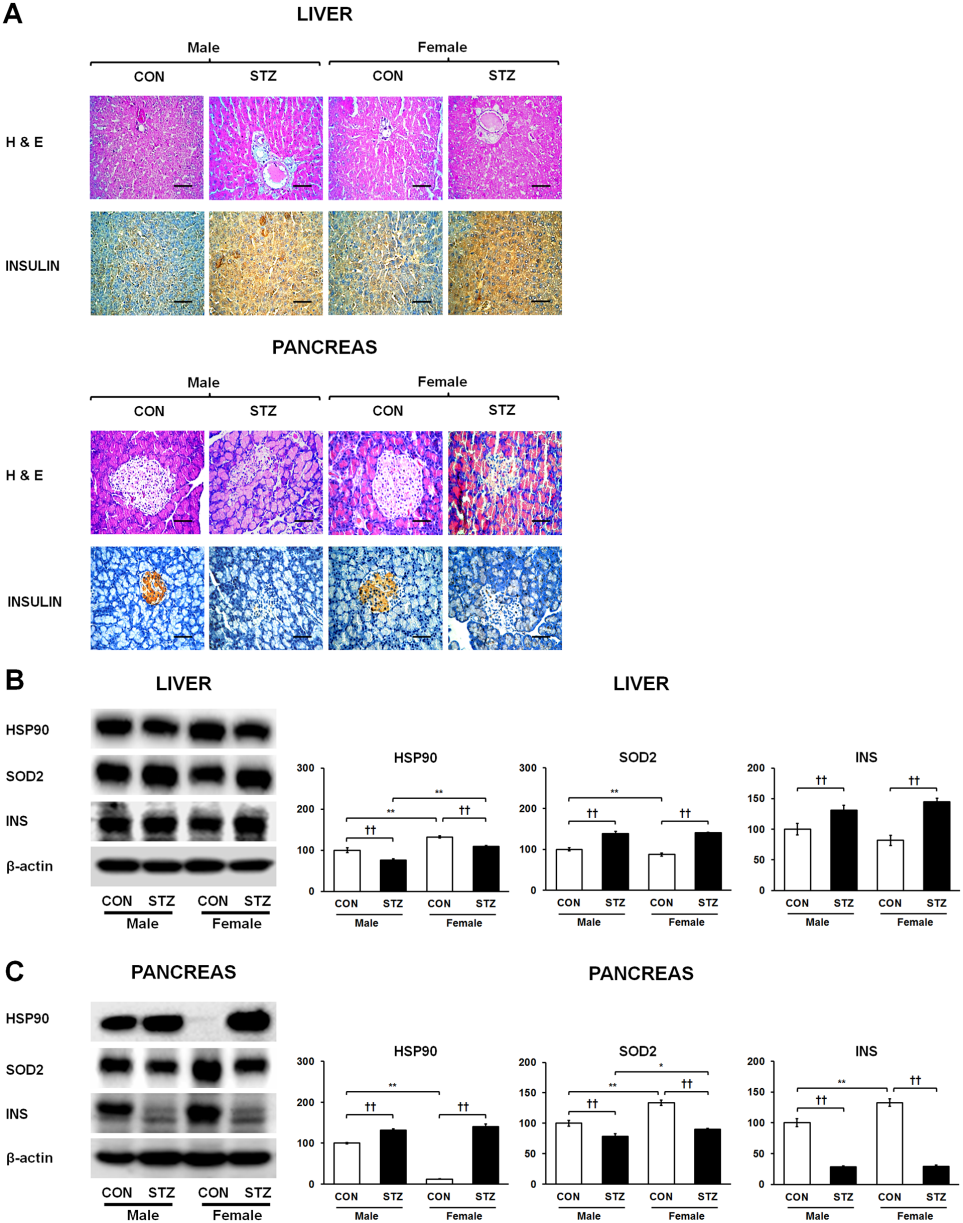
H&E staining profiles and immunohistochemistry of insulin in liver and pancreas (A), where representative photomicrographs are shown and immunohistochemical sections were counter-stained with haematoxylin (magnification X400 and scale bar represents 50 μm) as well as tissue-dependent protein expression levels of HSP90, SOD2, INS in liver (B) and pancreas (C), respectively. Data are representative of three independent experiments. Statistical significance between male and female rats was calculated by One-way ANOVA followed by Tukey’s post hoc tests, where *p*-value is *p<0.05 and **p<0.01, and significance between controls and diabetics is represented by ^†^ *p*<0.05 and ^††^ *p*<0.01.

To elucidate STZ-induced apoptosis and necrosis in a tissue-specific manner as well as understand the extent of inflammation-mediated injury between the diabetic liver and pancreas, we investigated protein levels of poly (ADP-ribose) polymerase 1 (PARP-1), ATP synthase subunit beta (ATP5B), and tumor necrosis factor receptor superfamily member 6 (CD95) in both tissues. Expression of ATP5B was up-regulated while PARP-1 expression showed no significant changes in the diabetic liver (Fig 4C). PARP-1 was strongly up-regulated along with consequent depletion of ATP5B in the diabetic pancreas (Fig 4D). Furthermore, protein expression levels of CD95 were increased and decreased in the STZ-induced diabetic liver and pancreas (Fig 4C and 4D), respectively. Histopathological analysis revealed that the diabetic liver showed injury associated with fibrosis. The pancreas of diabetic rats showed β-cell damage along with islet deformation and necrosis (Fig 5A).

### Effect of tissue-dependent cytoprotective capacity on liver and pancreas of diabetic rats

To determine whether tissue-specific expression of chaperone and antioxidant defense proteins can lead to differences in cellular protection, we measured protein levels of heat shock protein 90 (HSP90) and Mn-superoxide dismutase (SOD2) in the STZ-induced diabetic liver and pancreas. Interestingly, HSP90 showed elevated expression in the diabetic pancreas along with reduced expression in the diabetic liver (Fig 5B and 5C). On the other hand, SOD2 expression was elevated in the diabetic liver while strikingly suppressed in the diabetic pancreas (Fig 5B and 5C). Thus, gain or loss of cytoprotective capacity against cellular stress induced by STZ may be related to tissue-specific differential expression of SPARC in the diabetic liver and pancreas.

### Tissue-dependent expression of cellular insulin (INS) in diabetic liver and pancreas

To further verify tissue-dependent expression of cellular insulin, we performed immunohistochemical analysis of insulin in the STZ-induced diabetic liver and pancreas. Interestingly, diabetic liver showed strong insulin antigen positivity, whereas weak insulin immunoreactivity was detected in the diabetic pancreas (Fig 5A), which is in agreement with the immunoblot results (Fig 5B and 5C). Therefore, the differences in cellular insulin levels may be correlated with tissue-specific regulation of SPARC.

### Identification of SPARC-interacting partner proteins

**Fig 6:**
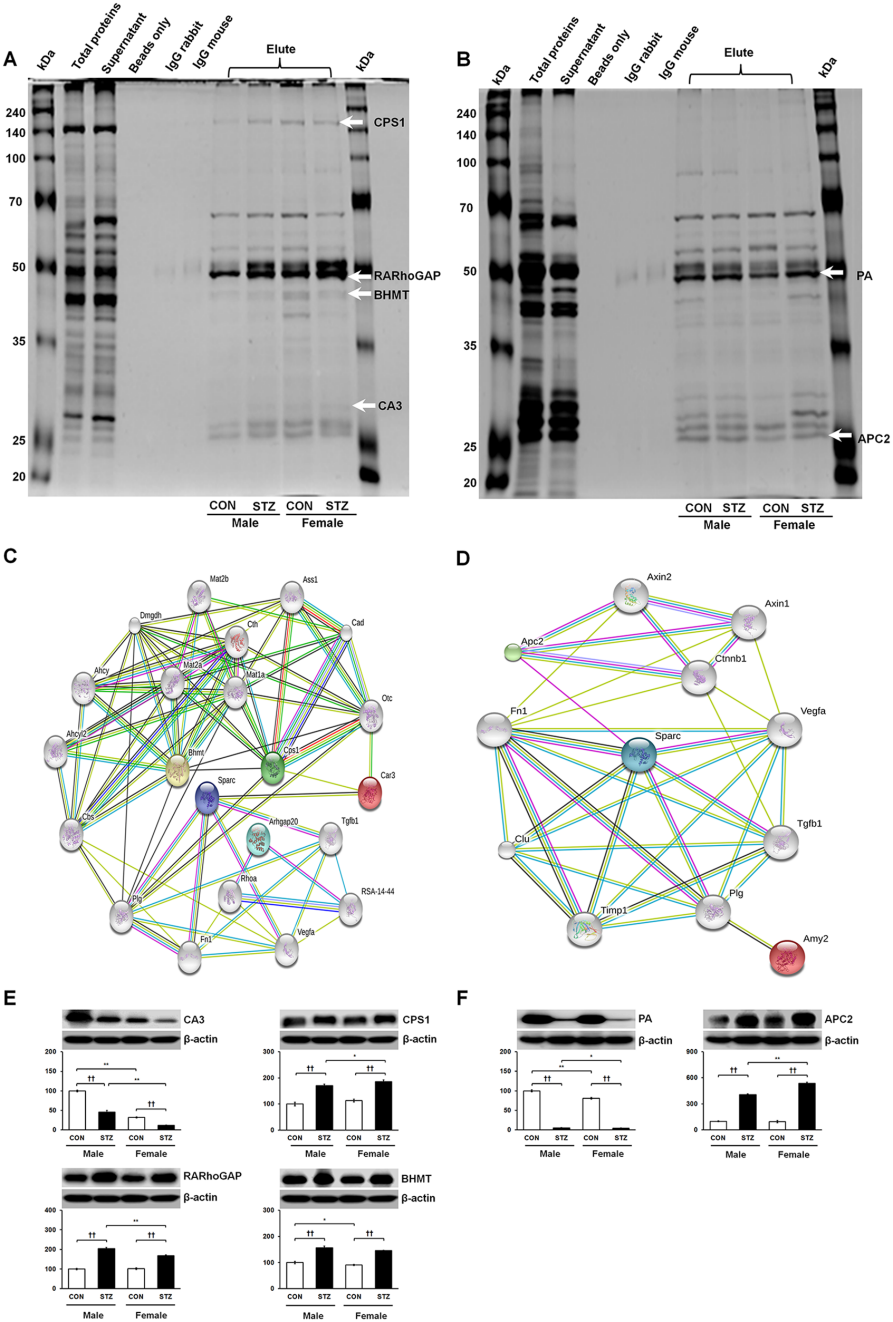
Representative silver-stained SDS-PAGE images of Co-IP samples in the liver (A) and pancreas (B) for identification of SPARC-interacting partner proteins. Proteins were identified by MALDI-TOF/MS analysis and are indicated by arrows along with their abbreviated names. Full names of proteins are presented in the abbreviations section. Physical interaction map analyzed by STRING in the liver (C) and pancreas (D). Expression patterns of SPARC-interacting partner proteins identified in the liver (E) and pancreas (F). Data are representative of three independent experiments. Statistical significance between male and female rats was determined by One-way ANOVA followed by Tukey’s post hoc tests, where *p-*value is *p<0.05 and **p<0.01. Significance between control and STZ-treated rats is represented by ^†^ *p*<0.05 and ^††^ *p*<0.01.

**Fig 7:**
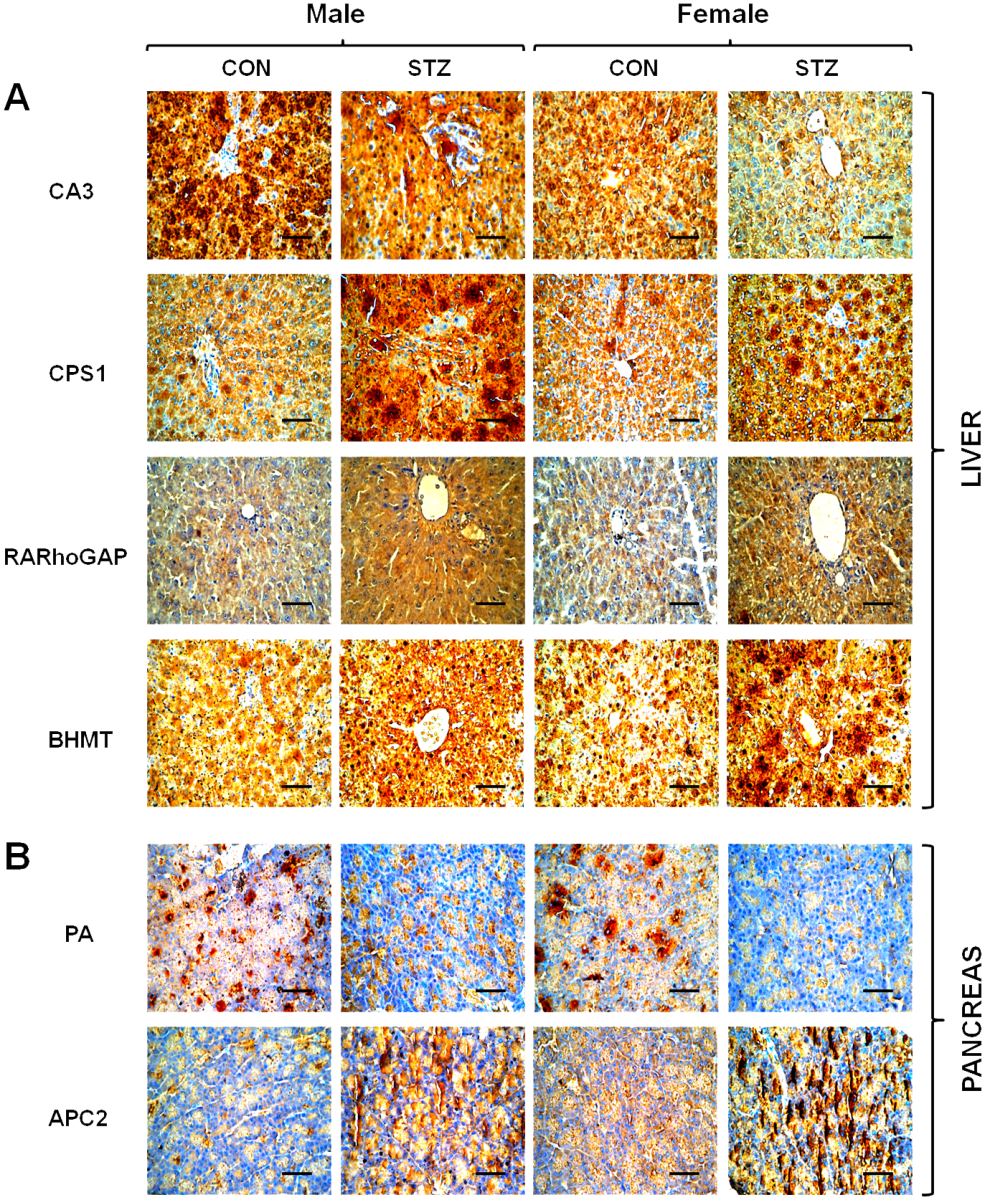
Immunohistochemistry of SPARC-interacting partner proteins (CA3, CPS1, RARhoGAP, and BHMT) in liver (A) as well as PA and APC2 in pancreatic tissue (B) from control and diabetic rats. Representative photomicrographs are shown from sections counter-stained with haematoxylin (magnification X400 and scale bar represents 50 μm).

We also performed Co-IP in order to isolate the interacting protein complexes of SPARC in the liver and pancreas. As shown in Fig 6A, PMF analysis revealed four novel interacting partners in the liver, including carbonic anhydrase 3 (CA3), carbamoyl-phosphate synthase (CPS1), Rho GTPase-activating protein 20 (RARhoGAP), and betaine-homocysteine S-methyl transferase (BHMT). Likewise, two novel SPARC-interacting partners were identified in the pancreas, including pancreatic alpha amylase (PA) and adenomatous polyposis coli protein (APC2) (Fig 6B). STRING network analysis predicted a direct association of SPARC/*Sparc* and CA3/*Car3*, which in turn proceeds through the series of interactions with BHMT/*Bhmt*, and CPS1/*Cps1* (Fig 6C) in the liver, whereas SPARC was indirectly interconnected with RARhoGAP/*Arhgap20*. STRING analysis also showed that SPARC/ *Sparc* interacted directly with APC2/*Apc2* in the pancreas as well as indirectly with PA/*Amy2* through intermediates (Fig 6D). Immunoblot analysis was performed for the identified SPARC-interacting protein partners, and found that CPS1, RARhoGAP, and BHMT were up-regulated while CA3 was down-regulated in the diabetic liver (Fig 6E). Meanwhile, APC2 expression was highly induced, whereas PA levels sharply declined in the diabetic pancreas (Fig 6F). Immunohistochemical analyses confirmed these immunoblot results by showing highly augmented immunoreactivity of CPS1, RARhoGAP, and BHMT in the diabetic liver (Fig 7A) as well as APC2 in the diabetic pancreas (Fig 7B). In contrast, CA3 and PA proteins showed less immunoreactivity in the diabetic liver and acinar region of the diabetic pancreas, respectively, as in the immunoblot results (Fig 7A and 7B).

### Pathway analysis and network generation

**Fig 8:**
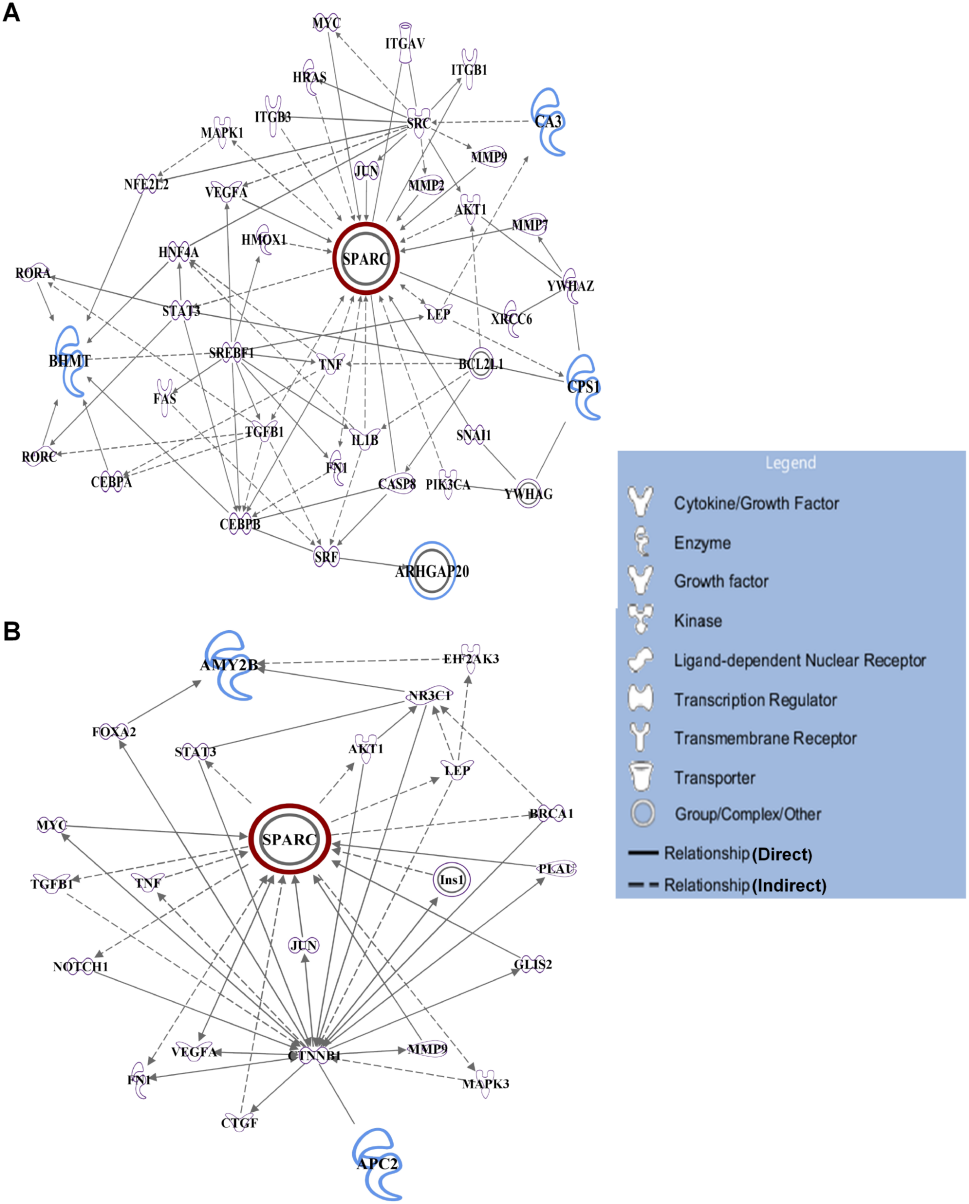
Functional interaction map generated by using Ingenuity Pathway Analysis (IPA). Tissue-specific prediction of SPARC-interacting protein network in the liver (A) and pancreas (B). Each node represents a protein and its biological relationship with other proteins is represented by a line or edge (either solid or dotted). Solid lines indicate direct relationships between proteins; dotted lines indicate indirect functional relationships. Nodes have different shapes to represent different functional classes as indicated in the legend within the figure. Nodes with bold outline represent SPARC (red color) and its interacting partner proteins (blue color) in the liver (BHMT, CA3, CPS1, and RARhoGAP/ARHGAP20) and pancreas (AMY2/PA and APC2). Proteins or nodes with no color were undetected in the study but have been incorporated by IPA to produce a highly connected network. A full list of cellular functions given by IPA for the molecular networks is provided along with statistical evidence in S1 and S2 Tables. Cellular functions involving only SPARC and its interacting proteins in the liver and pancreas were extracted from the main lists and given in S3 and S4 Tables, respectively.

To identify the cellular functional processes involving SPARC partner proteins in the liver and pancreas, we performed functional bioinformatics analysis using Ingenuity Pathway Analysis (IPA). The graphical representation of functional networks/pathways for SPARC and the identified interacting partner proteins corresponding to liver and pancreas are presented in Fig 8A and 8B, respectively. A full list of cellular functions derived from IPA along with statistical evidence for each function is provided in S1 and S2 Tables. Top three cellular functions were determined based on the most significant *p*-values. With respect to the pathway associated with liver-specific SPARC and its interacting proteins (Fig 8A), the top three cellular functions were associated with cell death and survival, cellular development, as well as cellular growth and proliferation. Interestingly, SPARC and CA3 were significantly mapped to the top three cellular functions. On the other hand, the top three cellular functions given by IPA for the molecular network of SPARC and its protein partners in the pancreas (Fig 8B) were embryonic, organismal, and cellular development. Of note, SPARC and APC2 were significantly mapped to the top three cellular functions. To get a better understanding of the cellular processes of SPARC and its interacting proteins in the liver and pancreas, cellular functions involving only those proteins were extracted from the entire list of functions and provided in S3 and S4 Tables. Functional annotations of SPARC and its partner proteins in the liver (CA3, CPS1, RARhoGAP/ARHGAP20, and BHMT) found with Ingenuity as shown in S3 Table, were interesting in light of the significant functionalities implicated in determining cell fate, like cellular function and maintenance, growth, differentiation, proliferation, cell death and survival, apoptosis, fibrosis, fibrogenesis, wound healing, regeneration of liver, inflammatory response, free radical scavenging, and glucose metabolism disorder. The overlap of these cellular functions entitled for SPARC and its interacting proteins in the liver may suggest cellular survival. Most of the cellular functions of SPARC and its partner proteins in the pancreas (PA/AMY2B and APC2) which were significantly annotated by IPA (S4 Table), appeared to be associated with chronic inflammatory conditions and related pathologies, like inflammatory and immune responses, differentiation, expansion, and infiltration of leukocytes, necrosis, amyloidosis, diabetes mellitus, non-insulin dependent diabetes mellitus, invasion, organ degeneration, adhesion, and arrest in cell cycle progression. These findings may implicate tissue-dependent regulation of SPARC and its interacting partners in the liver and pancreas. However, results of these pathway analyses were preliminary, and further studies are warranted to substantiate the cellular functions of SPARC and its interacting proteins in either organ.

## Discussion

In the present study, we examined the opposite expression of SPARC in the liver and pancreas of STZ-induced diabetic rats. Upon diabetes induction, SPARC might play distinct roles in hepatic and pancreatic tissues in terms of tissue remodelling, fibrogenesis, inflammation, apoptosis, and cytoprotective machinery. The most remarkable outcome of this study is that increased expression of SPARC in the diabetic liver may mediate recovery from injury, as one of the main functions of SPARC is tissue remodelling. The implication of SPARC in diabetic liver may be correlated with a causal role of SPARC in T2DM and obesity. More importantly, elevated plasma SPARC levels are potentially linked to the pathogenesis of T2DM [15], and inflammation in gestational diabetes mellitus [25]. Furthermore, higher expression of SPARC in adipose tissues is associated with obesity in human individuals [14] and mouse models [26]. Reduction of SPARC expression in the diabetic rat pancreas may play a role in the initiation of pancreatic pathology after STZ treatment. Similarly, a recent study reported decreased SPARC expression in diabetic pancreatic islets as well as significant growth of SPARC-overexpressed β-cells, providing evidence of SPARC involvement in β-cell function as well as insulin secretion [21]. An inverse correlation was also reported between basal insulin levels and SPARC expression in pancreatic islets, in contrast to adipose tissue.

The opposite expression of SPARC in the STZ-induced diabetic liver and pancreas prompted us to concentrate on several pro-inflammatory cytokines that are usual suspects in response to diabetic induction. Growing evidence indicates that activated immune cells are the principal sources of pro-inflammatory cytokines, which include TNFα, IL-1, IL-6, MPO, and CXCR2 [27], and their elevated levels are reported in connection with both T1DM and T2DM [28–32]. It is well known that C/EBPβ is a crucial transcription factor involved in the regulation of its target inflammatory mediators or cytokines such as TNFα, IL-1, IL-6, IL-8 [33, 34], and the acute phase proteins such as CRP and FN [33, 35]. In the current study, C/EBPβ expression was reduced in diabetic liver while increased in the diabetic pancreas. Therefore, C/EBPβ may play a crucial role in SPARC expression in STZ-induced diabetes. In fact, C/EBPβ is negatively modulated by SPARC during adipocyte differentiation [36], which suggests that up-regulated SPARC correlated with reduced expression of C/EBPβ in the STZ-induced diabetic liver. Importantly, C/EBPβ expression in the diabetic pancreas accelerates β-cell failure by enhancing susceptibility to endoplasmic reticulum (ER) stress [37]. Taken together, C/EBPβ may be positively associated with TNFα and its different regulatory activities in both diabetic tissues. This evidence along with the up-regulation of pro-inflammatory cytokines and acute phase molecules in the diabetic pancreas suggest that inflammation was more severe in diabetic pancreas than in the diabetic liver (Figs 3 and 4). In support of our result, previous studies have shown that TNFα strongly suppresses secretion of SPARC while expression of SPARC inhibits production of IL-6 [38, 39]. According to our observations, reduced SPARC expression in the diabetic pancreas implies that SPARC may be correlated with negative regulation of C/EBPβ, TNFα, *Il6*, CRP, and *Fn1* expression. Indeed, plasma levels of CRP and FN are elevated in patients with T2DM [40, 41]. Collectively, the opposite expression of selected inflammatory proteins or genes may be associated with tissue-specific expression of SPARC between the STZ-induced diabetic liver and pancreas.

Regardless of inflammatory status, concomitant expression of MCP-1, iNOS, NFκB, *Il1b*, and Cyt C in the diabetic liver and pancreas depicts a sequence of tissue-specific pathological changes leading to either resolution of inflammation in the liver or disorganization of pancreatic β-cells. This scenario may explain why iNOS has a protective effect on hepatic fibrosis in mice [42]. Conversely, β-cell-specific activation of the NFκB-iNOS-NO signaling pathway is involved in the progressive loss and apoptosis of β-cells in T1DM [43].

Next, we dissected the correlation of SPARC expression with fibrosis *versus* inflammation in the STZ-induced diabetic liver and pancreas. SPARC is also known to regulate TGF-β1 signaling [44]. Expression of SPARC is increased in hepatic fibrosis due to activation of TGF-β1, a profibrotic cytokine [18] with anti-inflammatory [44] and immunosuppressive properties [45]. This provides a basis that SPARC and TGF-β1 may be involved in minimizing inflammation. Risk of STZ-induced pancreatic β-cell damage has been associated with increased TNFα production. As TNFα and TGF-β1 have antagonistic functions in tissue repair and matrix remodelling [46], TGF-β1 has the ability to down-regulate TNFα [44] for tissue repair. Therefore, we speculate that SPARC at the onset of diabetes may be associated with regulation of *Tgfb1* as well as *Tgfb1*-mediated TNFα production. Meanwhile, SPARC-deficiency is associated with accelerated inflammatory infiltration, immune cell migration, disrupted fibrosis, aggressive inflammation, and inability to down-regulate TNFα due to impaired *Tgfb1* responses [44]. Aberrant inflammation in the diabetic pancreas may be susceptible to down-modulation of *Tgfb1*, which may be insensitive to reduction of TNFα expression. Simultaneously, severe immune cell infiltration of *Mpo* and *Cxcr2* along with elevated levels of *Fn1* might have antagonized damage in the diabetic pancreas. Increased expression of *Tgfb1* in the diabetic liver may be associated with regulation of TNFα production. The inverse relationship between *Tgfb1* and *Fn1* in the diabetic liver may positively regulate fibrosis as observed in fibronectin-deficient mice [47].

Although we cannot exclude the possibility that inflammation occurred in the male diabetic liver, as indicated by increased levels of *Mpo* and *Cxcr2*, increased modulation of *Tgfb1* with less up-regulated TNFα might have compensated for hepatic inflammation and promoted fibrogenesis. Taken together, SPARC may be associated with cross-regulation of TNFα and *Tgfb1* in inflammatory infiltration *via* its ability to preserve or distort *Tgfb1* activity towards anti-inflammation, fibrotic outcome, and remodelling. This suggests that SPARC expression may be related to different pathological settings in the STZ-induced diabetic liver and pancreas.

We next investigated apoptotic machinery and cellular energy balance between the STZ-induced diabetic liver and pancreas. Pancreatic damage may prevent CD95-mediated apoptosis which leads to PARP-1 up-regulation along with reduction of ATP5B expression [48]. Indeed, biopsy studies of patients with T2DM are found to exhibit increased expression of PARP-1 in skin [49], and decreased expression of ATP synthase in skeletal muscle [50]. Thus, low ATP levels may mean that necrosis, which is accelerated by TNFα [48], is one of the possible reasons for inhibition of SPARC in the diabetic pancreas. In contrast to the diabetic pancreas, PARP-1 was not up-regulated during CD95-mediated apoptosis in the diabetic liver and prevented ATP depletion by restoring high levels of ATP, thereby ensuring apoptosis [48]. Collectively, we postulate that a relationship may exist between CD95-mediated energy dependent-apoptosis and SPARC expression in the diabetic liver. It is likely that differential expression of SPARC between the diabetic liver and pancreas may be affected by the mode of cell death.

Further, we demonstrated the importance of cytoprotection for determining differences in SPARC expression between the diabetic liver and pancreas. The up-regulation of the HSP90 in the STZ-induced pancreas [51] appears to confer inadequate protection for β-cells. In fact, increased expression of HSP90 is observed in skeletal muscle biopsies of T2DM subjects [52]. Moreover, SOD2 expression was significantly reduced in the STZ-induced pancreas. Elevated levels of SOD2 in the STZ-induced diabetic liver are indicative of adequate antioxidant capacity [53], even though reduced HSP90 expression was observed. Thus, the diabetic liver may have eventually acquired sufficient protection from hepatic injury during STZ treatment. Further, it was demonstrated that SPARC expression is attenuated during chronic pancreatitis [20]. Impaired cytoprotective machinery and tissue-specific preservation of stress and inflammation may explain the depleted levels of SPARC in the STZ-induced diabetic pancreas.

Exceptionally, we found elevated levels of insulin in the diabetic liver as compared to non-diabetic liver. The possible increase of insulin in diabetic liver may be due to the presence of extra pancreatic insulin-producing cells in liver especially during diabetes and hyperglycemic states [54]. In fact, insulin mRNA, proinsulin, and insulin-positive cells are enriched in the liver of STZ-induced diabetic and obese murine models [54]. Intriguingly, increased and attenuated immunoreactivity of insulin may be correlated with positive and negative regulation of SPARC in the STZ-induced diabetic liver and pancreas, respectively. Furthermore, inhibition of insulin secretion during STZ-induced chronic pancreatic inflammation may be associated with attenuated expression of SPARC and thus mediated dysregulation of β-cell function in the diabetic pancreas.

To interpret the sex-dimorphic regulation of inflammatory molecules, we compared genes/proteins showing gender-specific expression patterns in diabetic rats. Non-immunological factors such as sex hormones were most likely responsible. When we assessed diabetic status based on sex-dependent plasma insulin levels, female rats showed greater impairment of insulin secretion than males. Estrogen is related to the suppression of TNFα, IL-1β, IL-6, MCP-1, and iNOS in different cell types of chronic inflammatory diseases [55], MPO in diabetic liver [56], and CXCR2 in peripheral blood monocytes during atherogenesis [57]. For example, a differential regulation of these molecules in male and female diabetic liver may be related to the different hormonal status because estrogens have protective effects against diabetes, playing a role in attenuation of cytokine and chemokine competence. Nonetheless, there was no significant sex-dependent regulation between the male and female diabetic pancreas upon STZ induction. However, the observed sex dimorphism in the diabetic liver does not fully explain the underlying sex differences in the progression of diabetes.

We also identified protein partners of SPARC and elucidated their effects on expression of SPARC in the STZ-induced diabetic liver and pancreas. SPARC interacted with CA3, and its reduced expression in the STZ-induced diabetic liver [58] may be involved in maintaining acid base homeostasis during tissue remodelling [59]. Collectively, reduced protein expression of CA3 in the diabetic liver may be associated with protection of cellular machinery from acid-base imbalances. Next, we identified the presence of another protein complex containing SPARC and BHMT. BHMT is up-regulated in the STZ-induced diabetic liver and involves in homocysteine homeostasis [60]. Moreover, BHMT is related with homocysteine at pathological concentrations that may be linked to inflammation, obesity, and diabetes [61], and involves the activation of NFκB/iNOS pathway [62]. Higher levels of homocysteine enhance ECM remodelling through matrix metalloproteinases and the plasminogen/plasmin system [63]. Activation of these matrix enzymes is regulated by SPARC [9]. Therefore, the STZ-induced diabetic liver might be protected from densely accumulated homocysteine-induced hepatic injury by accelerated ECM remodelling and rapid homocysteine clearance induced by overexpression of BHMT.

SPARC interacted with CPS1, which showed elevated expression in the diabetic liver [64]. Indeed, down-regulation of CPS1 results in the accumulation of uric acid [65], which may activate mitogen-activated protein kinase (MAPK) signaling pathway [66], and also cause inflammation, oxidative stress, insulin resistance, and metabolic syndrome, during the progression of liver disease [67], obesity, and T2DM [68]. In line with our findings, the link between SPARC and elevated CPS1 expression is necessary to prevent uric acid accumulation and protect against hepatic injury during STZ induction. In addition, our Co-IP data indicate that SPARC also interacted with RARhoGAP, and protein expression of RARhoGAP was highly elevated in the diabetic liver, as demonstrated by Gojo et al. [69]. Thus, RARhoGAP up-regulation may be associated with enhanced proliferative capacity and may provide additional support for apoptosis and cell survival through regulation of actin cytoskeleton assembly [70] in the STZ-induced diabetic liver. Nevertheless, RARhoGAP is found to be associated with renal inflammation in diabetic nephropathy *via* activation of NFκB [71], and also presumably activates JNK or p38 MAPK signaling [72]. In investigating SPARC-interacting protein partners in the pancreas, we identified pancreatic alpha amylase (PA) and adenomatous polyopsis coli protein 2 (APC2). Among these, PA was drastically down-regulated in the STZ-induced diabetic pancreas [73], and low pancreatic amylase levels are associated with chronic pancreatitis [74] more commonly in T1DM [75] than T2DM [76]. Indeed, amylase is known to be involved in carbohydrate digestion, and dysregulation of amylase is associated with reduced cellular calcium levels during STZ-induced diabetes [73]. Thus, altered calcium levels and enhanced inflammation along with dysregulated PA in the STZ-induced diabetic pancreas may affect expression of SPARC. Further studies are required to directly test this hypothesis.

Interestingly, APC2 as another partner protein of SPARC was strikingly up-regulated in the STZ-induced diabetic pancreas. In fact, overexpressed truncated APC protein not only disrupts or alters β-catenin/Wnt signaling [77] but also affects the rate of β-cell proliferation as well as the total mass of the β-cells, which can contribute to diabetes [78]. SPARC is involved in Wnt signaling [79], and therefore up-regulated APC2 in the diabetic pancreas may disturb β-catenin signaling and inhibit proliferation of β-cells. Indeed, activation of Wnt signaling is associated with inflammation in obesity [80], and diabetic retinopathy [81]. We suggest that the association of SPARC with its interacting protein partners may be linked to inflammatory responses underlying STZ-induced diabetes. A possible explanation for loss of SPARC in the diabetic pancreas may be the chronic inflammation induced by STZ, and overcoming this loss may be therapeutically relevant. Taken together, these findings support the idea that coordination between SPARC and its protein partners determines the preferential bioavailability of SPARC in the liver and pancreas during T1DM in terms of tissue remodelling and β-cell function, respectively.

**Fig 9:**
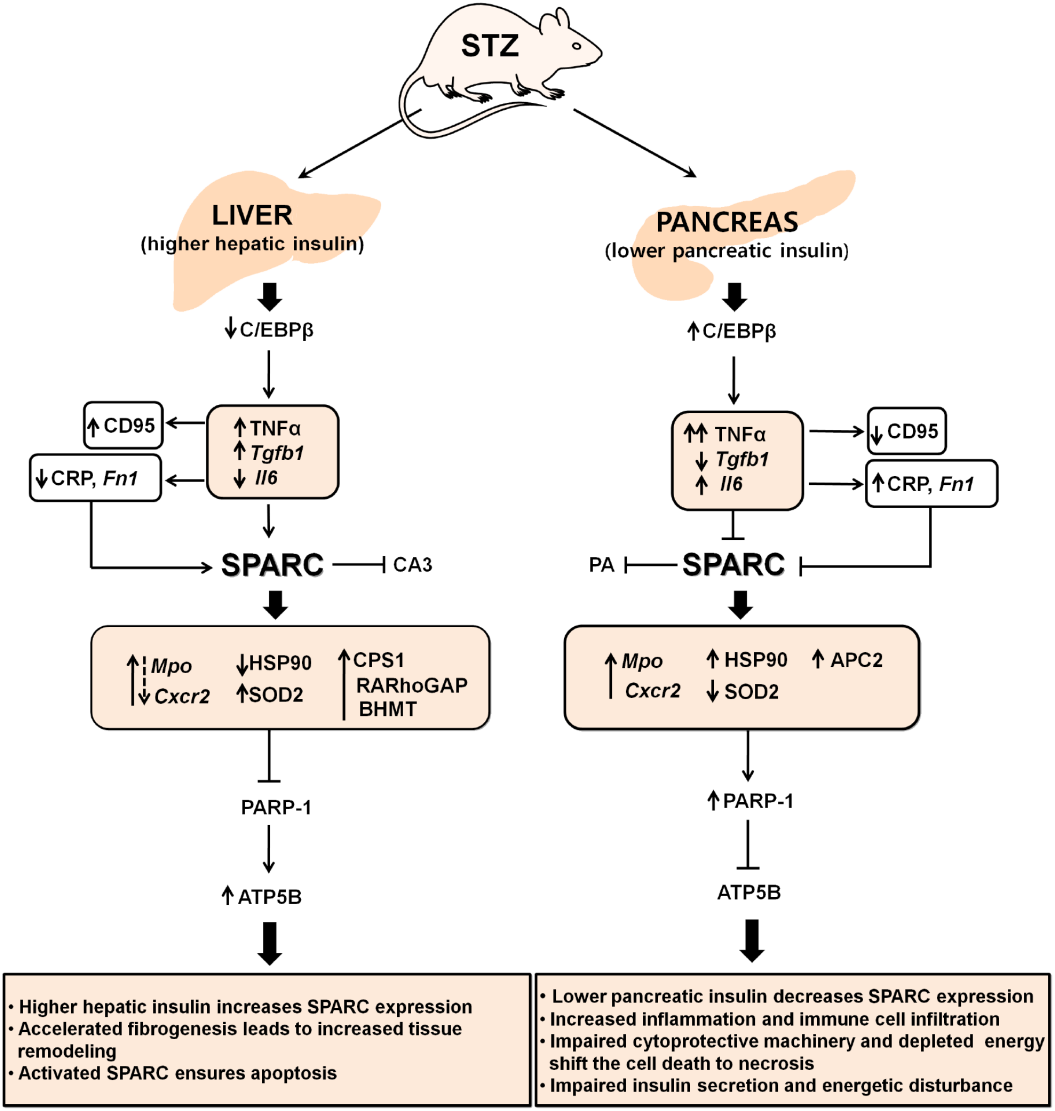
Summary scheme and brief description of roles of SPARC in the liver and pancreas of STZ-induced diabetic rats: (↓) stimulatory, (┴) inhibitory action.

In conclusion, the differences in SPARC expression between the diabetic liver and pancreas may be explained by variations in inflammation and immune cell infiltration, apoptosis, fibrogenesis, cytoprotective machinery, and cellular insulin levels. The proposed molecular mechanism underlying the differential regulation of SPARC in the STZ-induced diabetic liver and pancreas is illustrated in Fig 9. There are four possible explanations for the differential expression of SPARC in the liver and pancreas in STZ-induced diabetic rats: 1) SPARC may have anti-inflammatory and remodelling properties exerted through inflammation *versus* fibrotic outcome during STZ induction; 2) Activated SPARC may be a non-invasive biomarker of fibrogenesis; 3) SPARC may act as a determinant of apoptosis, cytoprotective machinery, and cellular energy balances; 4) SPARC may mediate insulin secretion and β-cell function. Although the mechanism is not completely understood, one must consider conditions of SPARC overexpression in the STZ-induced diabetic liver. Precise control of SPARC induction in the SPARC-deficient diabetic pancreas might participate in apoptosis, fibrogenesis, and tissue remodelling. Further studies are required to determine the regulation of tissue-specific SPARC and its pathology.

## Supporting Information

S1 Supporting Information Pathway analysis and network generation using IPA. (PDF)

S1 Table Ingenuity report for molecular network of SPARC and its interacting proteins in the liver. In this table, the cellular functions were ranked in a descending order (top to bottom) based on the *p*-value.
(XLS)

S2 Table Ingenuity report for molecular network of SPARC and its interacting proteins in the pancreas. In this table, the cellular functions were ranked in a descending order (top to bottom) based on the *p*-value.
(XLS)

S3 Table Cellular functions of SPARC and its interacting proteins in the liver. In this table, we represent the cellular functions involving SPARC and its interacting proteins in the liver were extracted from S1 Table.
(XLS)

S4 Table Cellular functions of SPARC and its interacting proteins in the pancreas. In this table, the cellular functions involving only SPARC and its interacting proteins in the pancreas were extracted from S2 Table.
(XLS)
