# Supplementary material for: Opposite Expression of SPARC between the Liver and Pancreas in Streptozotocin-Induced Diabetic Rats
Source: PLoS One. 2015 Jun 25;10(6):e0131189. doi: 10.1371/journal.pone.0131189 (PMC4481468; doi:10.1371/journal.pone.0131189)
Supplement: S1 Supporting Information — (PDF) [file pone.0131189.s001.pdf]

S1 Supporting information

Pathway analysis and network generation using IPA

IPA typically generates molecular networks/pathways or interaction maps and also assigns the most important functionalities to these networks. Independent network analyses were performed for SPARC-interacting proteins in the liver and pancreas. Protein accession numbers or protein IDs of detected SPARC and its interacting partners were uploaded into IPA to create tissue-specific biological networks in the liver and pancreas, respectively. Each protein identifier was mapped to its corresponding protein object in the Ingenuity Pathways Knowledge Base. SPARC-interacting proteins in the liver (BHMT, CA3, CPS1, and RARhoGAP/ARHGAP20) and pancreas (PA/AMY2B and APC2) were used as the starting point for algorithmically generating the respective biological networks based on their connectivity as established in the published literature. Restricting species to 'rat', tissue to 'liver' or 'pancreas', disease to 'metabolic disease', and allowing for findings to experimentally observed/highly predicted confidence levels, direct/indirect interactions, and exclusion of chemicals and drugs, interactions were added using the IPA Path Explorer application. Given the specified criteria, this application was used to: (i) add a number of extra molecules or relationships from the knowledge database, and (ii) find among the Ingenuity Knowledge Base and external databases for the shortest paths connecting the selected nodes through one or two IPA-derived intermediate molecules to explore putative biologic interactions. For example, SPARC was selected to node-A and BHMT was selected to node-B to generate biological interactions between SPARC and BHMT, and rest of the interactions were predicted for all probable combinations of SPARC (mapped always as central node) and its interacting proteins. The shortest paths by which SPARC and its interacting proteins were integrated required minimum one or two interconnecting nodes.

Thus, for majority of possible interactions in the network, SPARC was mapped as a central node connecting its interacting partners with multiple protein factors suggested by Ingenuity Knowledge Base, and external databases which resulted in generating a predicted molecular network. In the network diagrams, each node represents a protein and its biological relationship with other proteins is represented by a line (either solid or dotted). Nodes have different shapes to represent different functional classes. Nodes highlighted in bold outline represent SPARC (red color) and its interacting partner proteins (blue color) in the liver (BHMT, CA3, CPS1, and RARhoGAP/ARHGAP20) and pancreas (PA/AMY2B and APC2). Proteins or nodes with no color were undetected in the study but have been incorporated by IPA to produce a highly connected network. In addition, we subjected the molecular networks that were built by Ingenuity to functional analysis using the Overlay tool. As a result, IPA assigns bio-functional categories/cellular functions (along with statistical evidence) to the respective molecular networks, indicating the *p*-value ranges, and the molecules involved for each category. *p*-Value referred to a right-tailed Fisher's exact tests calculating that the set of proteins were associated with a specific disease/function by chance alone.
